# Morphological parameters of middle cerebral arteries associated with aneurysm formation

**DOI:** 10.1007/s00234-020-02521-w

**Published:** 2020-08-19

**Authors:** Wei Zhang, Juan Wang, Ting Li, Mingjin Mei

**Affiliations:** 1 Department of Radiology, Brain Hospital of Hunan Province, No. 427, Section 3, Furong Middle Road,Yuhua District, Changsha City, Hunan Province, China; 2 Department of Radiology, The Third Xiangya Hospital of Central South University, No. 138 Tongzipo Road, Yuelu District, Changsha, Hunan China; 3 GE Healthcare Medical Affair, 10/F GE Tower, No. 87 Hua Cheng Avenue, Pearl River New City, Tianhe District, Guangzhou, Guangdong China

**Keywords:** Middle cerebral artery, Morphological parameters, Intracranial aneurysm, CT angiography

## Abstract

**Purpose:**

The objective of this work was to investigate the correlation between morphological parameters of the MCA and the formation of aneurysms.

**Methods:**

MCA aneurysms were diagnosed in 122 cases using CT angiography (including 30 cases of M1 proximal aneurysms, 70 cases of M1 bifurcation aneurysms, and 22 cases of distal aneurysms). Images from these cases were retrospectively compared with images from 50 healthy controls. Morphological parameters including the angle of the MCA with the ICA (*α*) and the ACA (*β*) were evaluated in the three aneurysm groups and the control group; parent-daughter angles (*γ*_1_, *γ*_2_), bifurcation angles (*γ*_3_), bifurcation diameters, angle ratios, and branch diameter ratios were also compared between the bifurcation aneurysm group and the control group. The blood vessel parameters between the aneurysm groups and controls were analyzed statistically.

**Results:**

There was no statistically significant difference in *α* between the three groups of aneurysms and the control group (*P* = 0.381). In comparing *β* between the three groups of aneurysms and the control group, statistically significant differences were only observed between the MCA distal aneurysm group and the control group (*P* = 0.010). Compared with the control group, MCA bifurcation aneurysms were associated with larger *γ*_3_ and smaller *γ*_1_ and *γ*_2_ (*P* < 0.001). This resulted in significantly larger angle ratios in the MCA bifurcation aneurysm group (*P* < 0.001). For the diameter measurements, the bifurcation diameter of the MCA bifurcation aneurysms was significantly smaller (*P* = 0.001).

**Conclusion:**

The formation of MCA aneurysms is related to morphological parameters.

## Introduction

Intracranial aneurysms are characterized by a local abnormal expansion of an arterial wall. The incidence of intracranial aneurysms is relatively high, and approximately 50% of patients with subarachnoid hemorrhages caused by aneurysm rupture are left with severe disability; furthermore, there is a 23~35% mortality rate when aneurysms are left untreated [1, 2]. There are many reports on the mechanisms of intracranial aneurysm (IAs) formation, which are currently considered to be related to heredity, hemodynamics, hypertension, inflammatory response, and lipid metabolism disorders. There is evidence that genetic factors, such as genetic polymorphisms, play an important role in aneurysm formation. Wu et al. found that the LOXL2c.C133T mutation was a pathogenic gene by conducting complete exon sequencing of representative families with a history of IAs [3]. Theodotou et al. retrospectively analyzed the literature on cerebral aneurysm formation and genetic correlations from December 2008 to June 2015. Their work found that gene 9p21/CDKN2, mainly involved in vascular wall remodeling, had the strongest correlation with IAs. Other genes potentially related to aneurysm formation include EDNRA and SOX17 [4].

MCA aneurysms account for 20% of intracranial aneurysms, with a rupture rate of approximately 50% [5]. MCA aneurysms are usually found after rupturing. They often result in intracranial hemorrhage and have higher mortality and disability rates compared with other IAs [6]. Therefore, it is of great importance to effectively screen high-risk groups regularly for early diagnosis and treatment of MCA aneurysms.

MCA aneurysms can be divided into three types according to their location: (1) proximal aneurysms located in the MCA prior to the bifurcation of the M1 segment (2–7% of MCA aneurysms), (2) bifurcation aneurysms located in the first bifurcation of the MCA (80–85% of MCA aneurysms), and (3) distal aneurysms located in the M2 segment and beyond (2~6% of MCA aneurysms) [7–10].

Due to the high incidence of MCA bifurcation aneurysms, many studies have focused on the formation of aneurysms as a result of morphological factors. These studies have confirmed that the formation of these aneurysms is related to the diameter and angle of the MCA bifurcation [11, 12], but did not study whether aneurysm formation was related to the morphological angle of the MCA with the internal carotid artery (ICA) and anterior cerebral artery (ACA). In addition, the association between the formation of proximal and distal aneurysms of the MCA and the angle between the MCA and ICA and ACA has not been previously reported. The bifurcation has special hemodynamic characteristics associated with an increased risk of aneurysm formation [13].

The purpose of this work was to study whether the angle of the MCA with the ICA and ACA contributes to the formation of MCA aneurysms. In our study, the correlation between MCA geometry and aneurysm formation was investigated to provide references for aneurysm screening and risk assessment in high-risk patients.

## Methods

### Patient selection

**Fig. 1:**
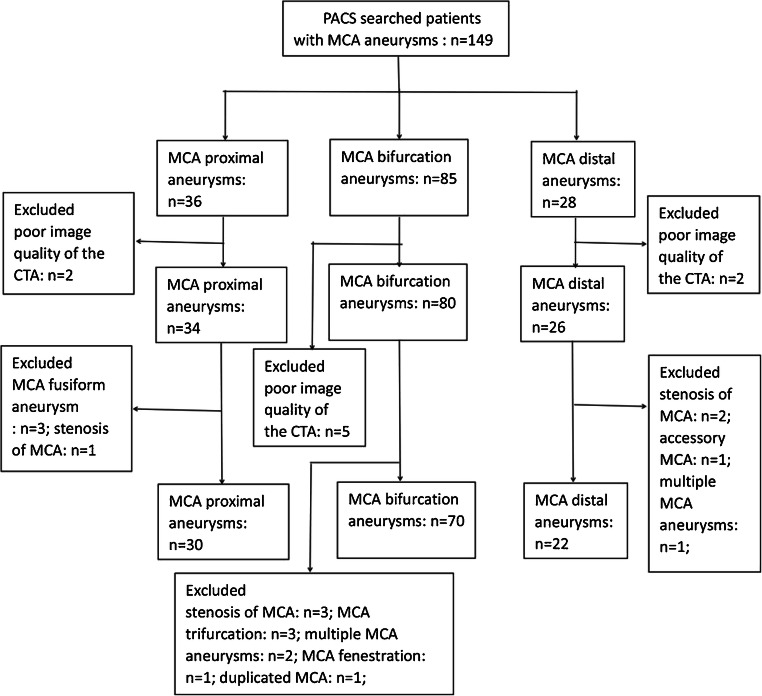
Flowchart for inclusion of patients with MCA aneurysms

This study was approved by the local medical ethics committee. Due to the retrospective nature of this study, informed consent was waived. A total of 122 patients with MCA aneurysms and 50 patients without aneurysms were enrolled from January 2014 to September 2019. The MCA aneurysm group was divided into three subgroups: (1) M1 segment proximal aneurysm (*n* = 30), (2) M1 segment bifurcation aneurysm (*n* = 70), and (3) distal aneurysm (*n* = 22). Inclusion criteria in the control group were absence of intracranial arteriovenous malformations, Moyamoya disease, subarachnoid hemorrhage, vasospasm, intracranial hemorrhage, or Circle of Willis loop aneurysms. The exclusion criteria of the aneurysm group were (1) fusiform aneurysm of the MCA or arteriovenous malformations, (2) stenosis of MCA vessels, (3) multiple MCA aneurysms, MCA trifurcation, early bifurcation, duplicated MCA, accessory MCA, or fenestration, and (4) poor image quality of the CTA examination. The flowchart of the patients with MCA aneurysms included in the analysis is shown in Fig. 1.

### Data acquisition

All subjects were scanned using three-dimensional CTA with 128-slice CT (Philips Ingenuity Core, scanning parameters: 120 kVp, 240 mAs, contrast material: iopromide 370 mg/ml, injection speed 5.0 ml/s, dose 50 ml) or 16-row CT (GE Bright Speed, scanning parameters: 120 kVp, 300 mAs, contrast material: iopromide 370 mg/ml, injection speed 4.0 ml/s, dose 60 ml). Images were transmitted to Philips Extended Brilliance Workspace for vascular reconstruction, bone removal and silhouette, and three-dimensional volume rendering (3D VR) and maximum-intensity projection (MIP). We measured by Philips Advanced Vessel Analysis Software. The vessel diameter was measured by using VR image, and the window setting for VR image was integrated based on the full width at half maximum on maximum intensity projection. Each dataset was manually measured by two primary physicians, who in turn performed each measurement twice. The average of these two measurements was used for analysis.

### Definition of morphological parameters

**Fig. 2:**
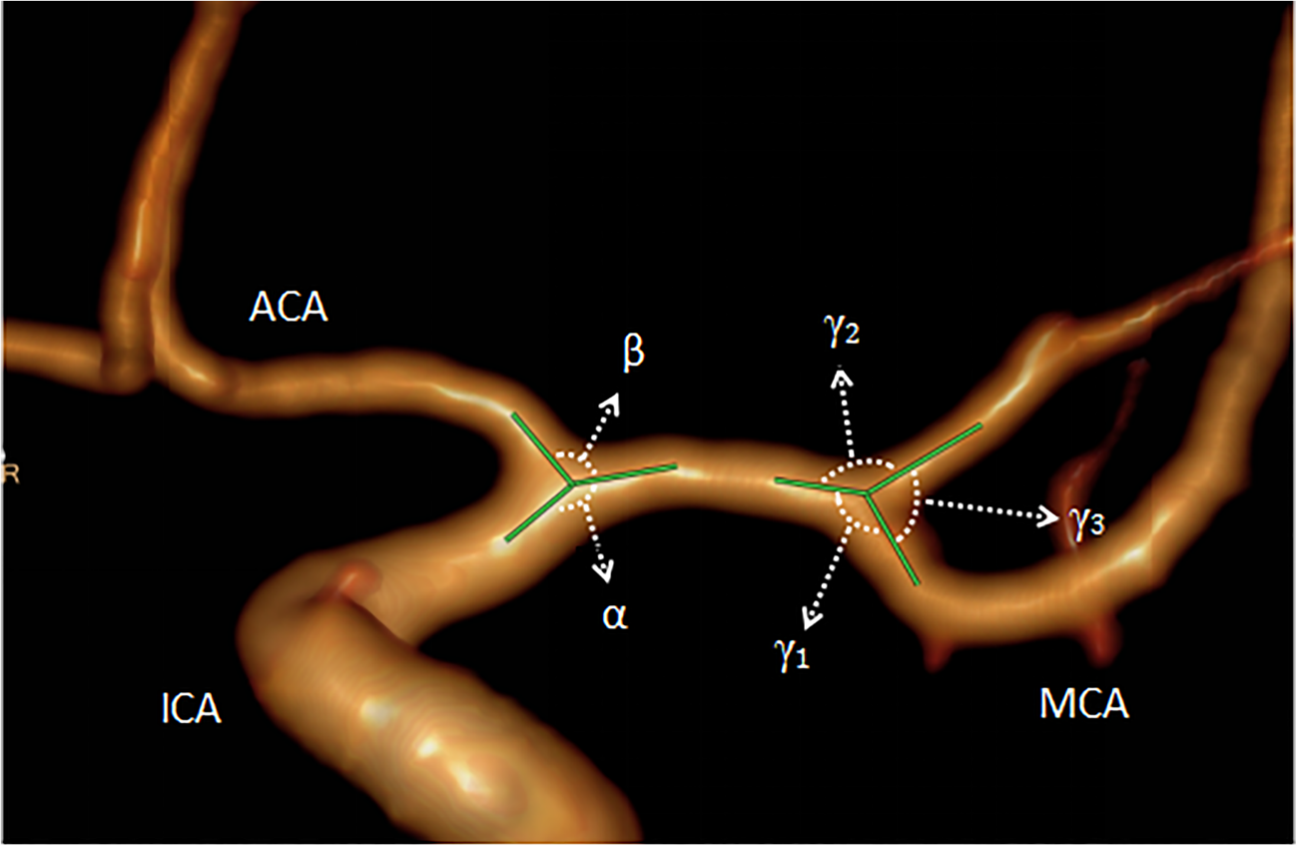
Angles and angle ratios were measured and defined as follows: ICA-MCA angle (*α*), ACA-MCA angle (*β*), *γ*_1_ between the larger daughter vessel and parent vessel, *γ*_2_ between the smaller vessel and parent vessel, *γ*_3_ between the larger daughter vessel and the smaller vessel.

**Fig. 3:**
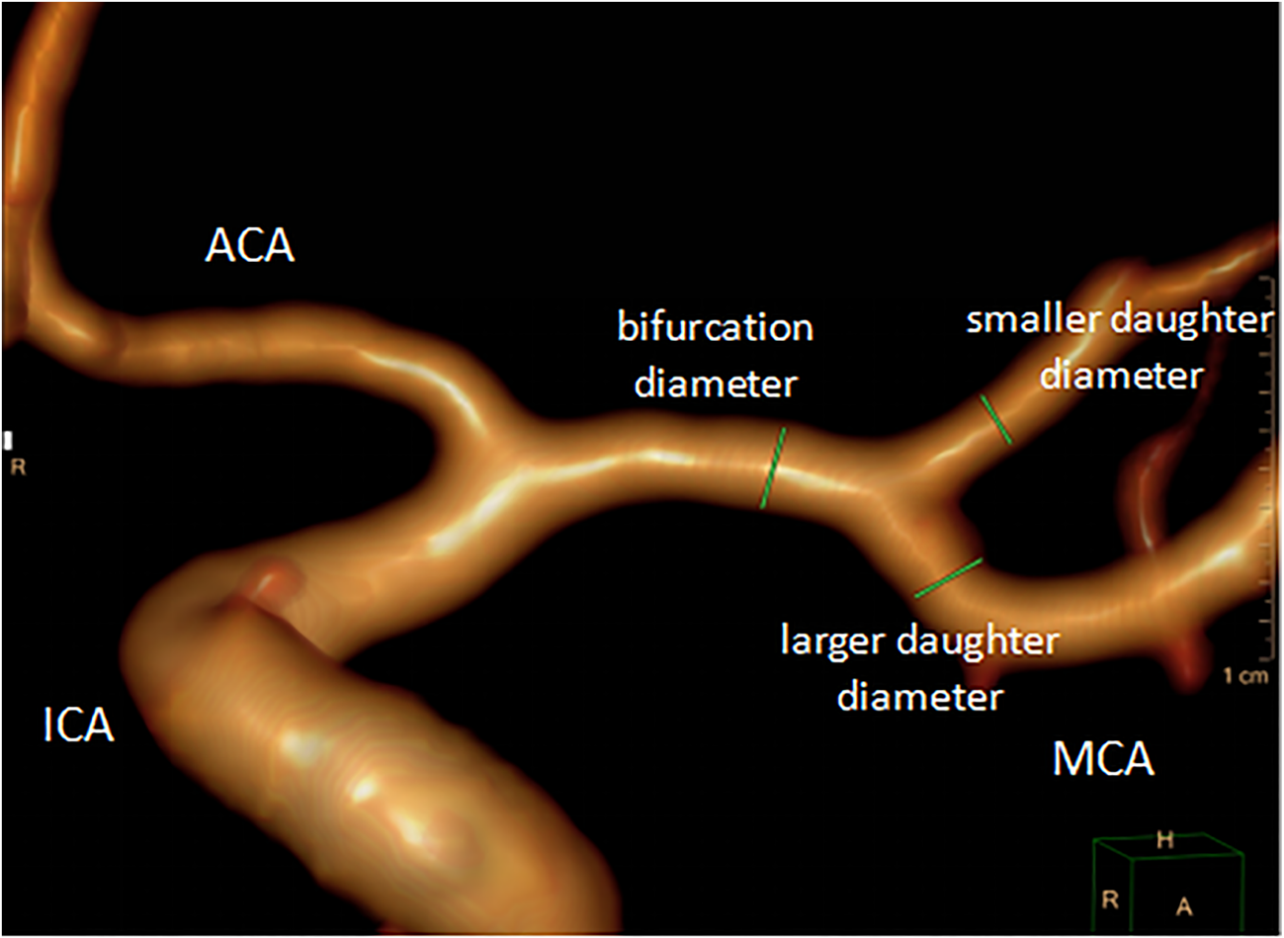
Diameter and diameter ratios were measured and defined as follows: the diameter of the M1 segment bifurcation; the branch diameter ratio was calculated by the ratio of the larger daughter diameter to the smaller daughter diameter

See Figs. 2 and 3 for definitions of morphologic measurements. All angles were measured on a three-dimensional longitudinal section of the center of the two vessels of interest. These measured angles included the following: between the MCA and ICA—*α*; between the MCA and ACA—*β*; bifurcation angle between the larger daughter vessel and parent vessel—*γ*_1_; between the smaller vessel and parent vessel—*γ*_2_; and between the larger daughter vessel and smaller vessel—*γ*_3_. Angle ratio was calculated as the ratio of larger parent-daughter angle to smaller parent-daughter angle. Diameter measurements were taken in the M1 segment bifurcation approximately 2 vessel diameters from the bifurcation [14]. The branch diameter ratio was calculated as the ratio of the larger daughter diameter to the smaller daughter diameter.

### Statistical analysis

In the control group and the bifurcation aneurysm group, *γ*_1_, *γ*_2_, *γ*_3_, the bifurcation diameter branch, and the diameter ratio were tested independently using a *t* test for normally distributed data; angle ratios were tested using a Wilcoxon rank-sum test for non-normally distributed data. The Kruskal-Wallis H test was used for *α* and *β* between the control group and the three groups of aneurysm, respectively. Receiver operating characteristic (ROC) curves of morphological parameters with statistical significance were obtained to determine cut-off values and then to evaluate the sensitivity and specificity of diagnosis. Age, sex, hypertension, diabetes, and hyperlipidemia were analyzed for differences between the control group and three aneurysm groups by using the *χ*^2^ test. Across all tests, *P* < 0.050 indicated a statistically significant difference. SPSS release 20.0 software (IBM Corp, Armonk, New York) was used for the statistical analysis.

## Results

The study included 33 males and 17 females in the control group. The mean (± standard deviation) age of the patients in the control group was 56.36 (± 13.51) years, ranging from 22 to 81 years. The three aneurysm groups included 46 males and 76 females. The mean age of the patients in the three aneurysm groups was 58.42(± 12.06) years, ranging from 25 to 88 years. There was no significant difference in age between the three MCA aneurysm groups and the control group. Compared with the control group, the number of females in aneurysm groups was significantly higher than the number of men (*P* = 0.001). And statistical analysis of the clinical factors of hypertension, diabetes, and hyperlipidemia is presented in Table 1.

**Table 1:** Summary of all patient´s clinical data

	Control (*n* = 50)	MCA proximal aneurysms(*n* = 30)	MCA bifurcation aneurysms (*n* = 70)	MCA distal aneurysms(*n* = 22)	*F*/?^2^	*P* value
Age (years)	56.36 ± 13.51	54.53 ± 12.05	60.42 ± 10.23	57.32 ± 16.05	1.198	0.119
Gender M/F	33:17	16:14	24:46	6:16	5.401	0.001
Diabetes(N/Y)	33:17	16:14	36:34	11:11	3.026	0.388
Hypertension(N/Y)	19:31	12:18	19:51	7:15	2.324	0.508
Hyperlipidemia (N/Y)	28:22	14:16	35:35	11:11	0.761	0.859

*M* male, *F* female, *N* without, *Y* with

**Fig. 4:**
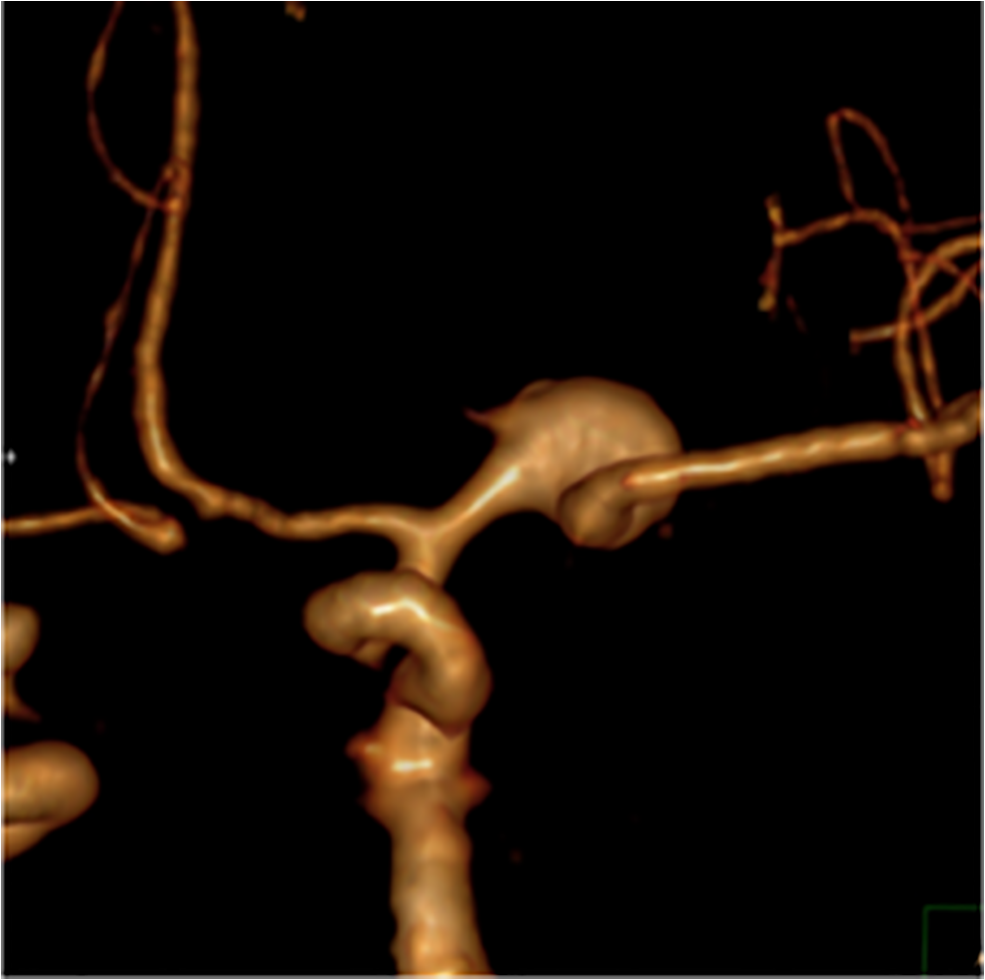
An example of an anterior view of a 3D rendered MCA proximal aneurysm is shown

**Fig. 5:**
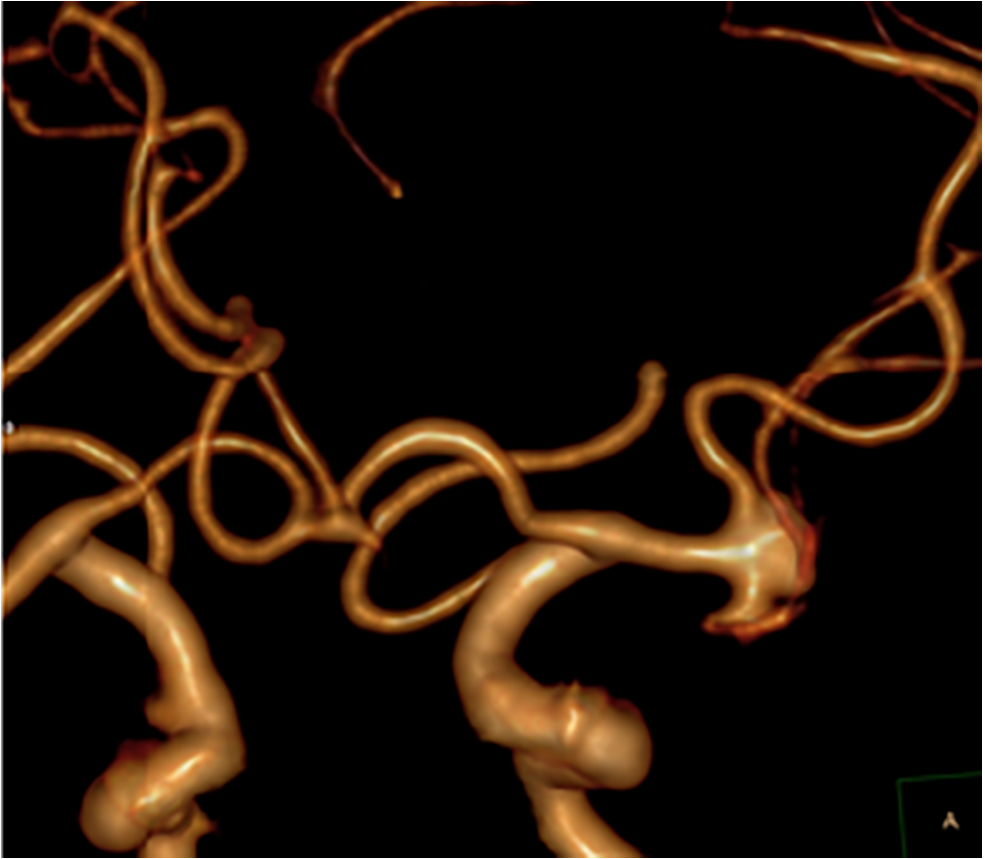
An example of an anterior view of a 3D rendered MCA bifurcation aneurysm is shown

**Fig. 6:**
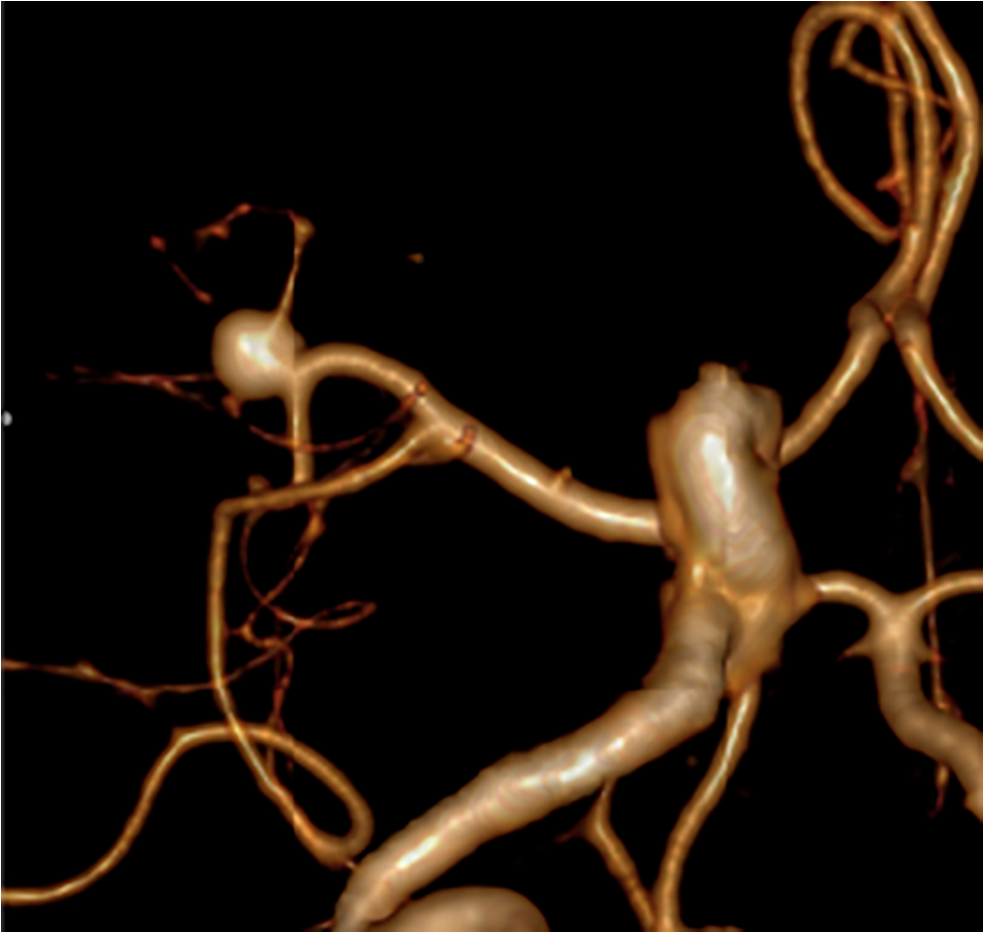
An example of a posterior view of a 3D rendered MCA distal aneurysm is shown

3D renderings of each type of aneurysm are shown in Figs. 4, 5, and 6 and depict excellent delineation of the vessel and aneurysm wall in all subtypes.

**Fig. 7:**
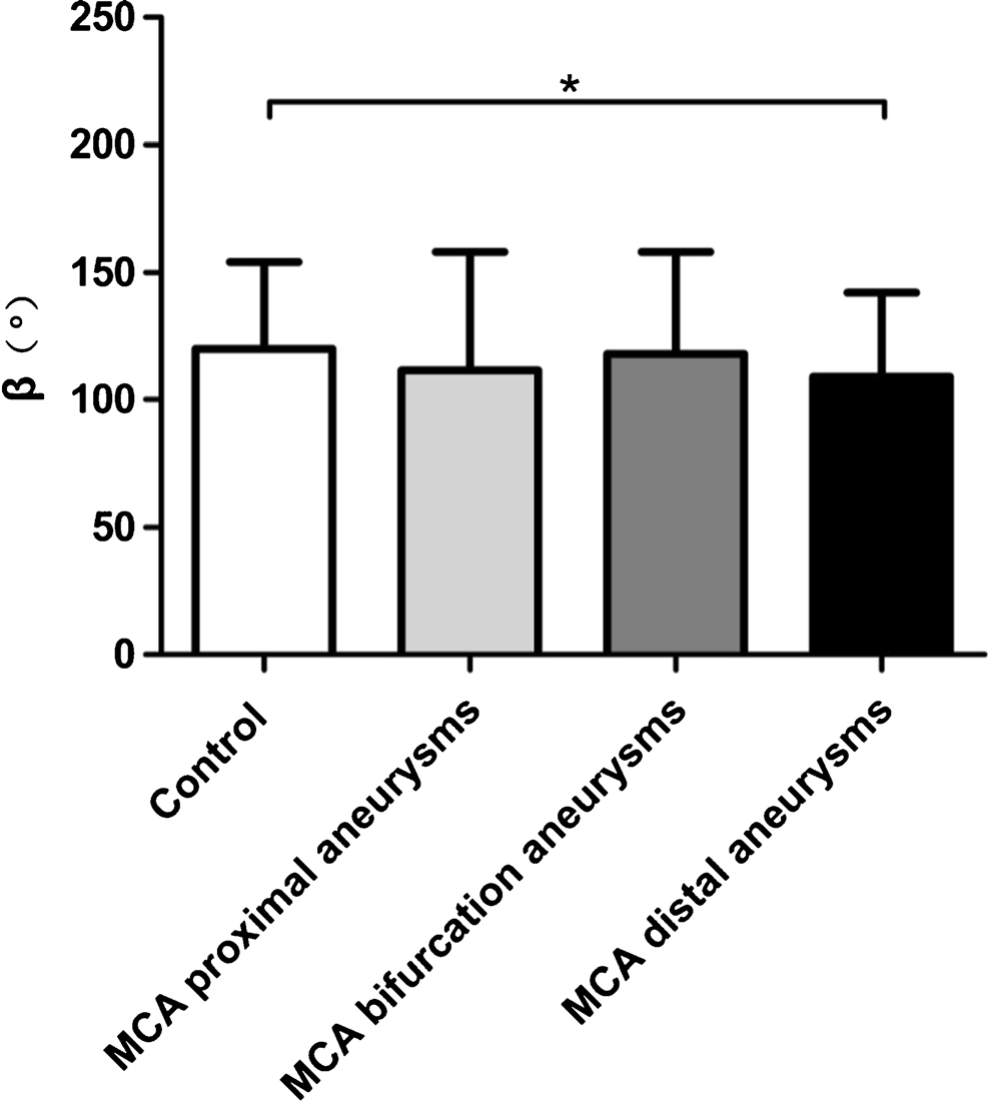
The MCA-ACA angle (*β*) between the three aneurysm groups and the control group is plotted as bar graphs with standard deviation error bars. *p < 0.05, **p < 0.001

At the MCA-ACA junction (Table 2, Fig. 7), there was no statistically significant difference in *α* between the three groups of aneurysms and the control group (*P* = 0.381). In comparing *β* between the three groups of aneurysms and the control group, statistically significant differences were only observed between the MCA distal aneurysm group and the control group (median 109.00° (19.00°) vs. 120.00° (19.25°), *P* = 0.010).

**Table 2:** Comparison of vascular morphological parameters between three groups of MCA aneurysms and the control group

	Control (*n*=50)	Aneurysms(*n* = 122)			*H* value	*P* value
		MCA proximal aneurysms (*n* = 30)	MCA bifurcation aneurysms (*n* = 70)	MCA distal aneurysms (*n* = 22)		
*α*(°)	134.50 (17.25)	139.50 (23.50)	140.00 (21.25)	143.00 (16.00)	3.072	0.381
*β*(°)	120.00 (19.25)	111.50 (29.25)	118.00 (28.25)	109.00 (19.00)	8.047	0.045

*α* and *β* using Kruskal-Wallis *H* test. Shown are median (interquartile range)

**Fig. 8:**
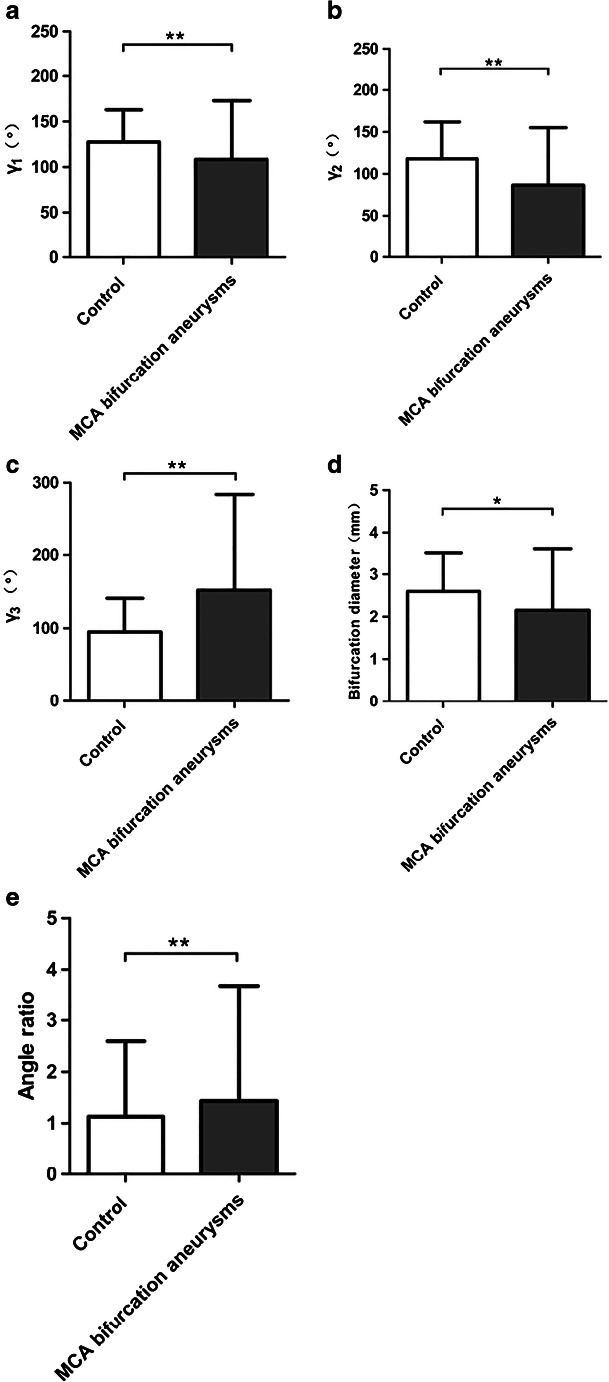
Results of *γ*_1_ (**a**), *γ*_2_ (**b**), *γ*_3_ (**c**), the bifurcation diameter (**d**), and the angle ratio (**e**) between the MCA bifurcation aneurysm group and the control group are plotted as bar graphs with standard deviation error bars. *p < 0.05, **p < 0.001

Statistical analysis results comparing the MCA bifurcation aneurysm group and the control group are shown in Table 3 and Fig. 8. Compared with the control group, MCA bifurcation aneurysms were associated with larger *γ*_3_ (mean 143.74° ± 36.57° vs. 95.45° ± 19.55°, *P* < 0.001) and smaller *γ*_1_ (mean 109.59° ± 30.68° vs. 128.78° ± 16.54°, *P* < 0.001) and *γ*_2_ (mean 87.21° ± 27.46° vs. 117.33° ± 22.16°, *P* < 0.001). This resulted in significantly larger angle ratios in the MCA bifurcation aneurysm group (mean 1.46 ± 0.77 vs. 1.13 ± 0.27, *P* < 0.001). For the diameter measurements, the bifurcation diameter of the MCA bifurcation aneurysms was significantly smaller (mean 2.18 ± 0.56 vs. 2.53 ± 0.53, *P =* 0.001), and there was no statistical difference in the branch diameter ratio (*P* = 0.920).

**Table 3:** Comparison of vascular morphological parameters between MCA bifurcation aneurysms and the control group

	Control (*n* = 50)	MCA bifurcation aneurysms(*n* = 70)	*t/Z* value	*P* value
*γ*_1_(°)^a^	128.78 ± 16.54	109.59 ± 30.68	4.336	< 0.001
*γ*_2_(°)^a^	117.33 ± 22.16	87.21 ± 27.46	6.221	< 0.001
*γ*_3_ (°)^a^	95.45 ± 19.55	143.74 ± 36.57	− 8.136	< 0.001
Bifurcation diameter(mm)^a^	2.53 ± 0.53	2.18 ± 0.56	3.374	0.001
Angle ratio^b^	1.13(0.27)	1.46(0.77)	− 4.000	< 0.001
Branch diameter ratio^a^	1.49 ± 0.45	1.49 ± 0.38	0.101	0.920

^a^*t* test, shown are mean and standard deviation

^b^Wilcoxon rank-sum test, shown are the median (interquartile range)

**Fig. 9:**
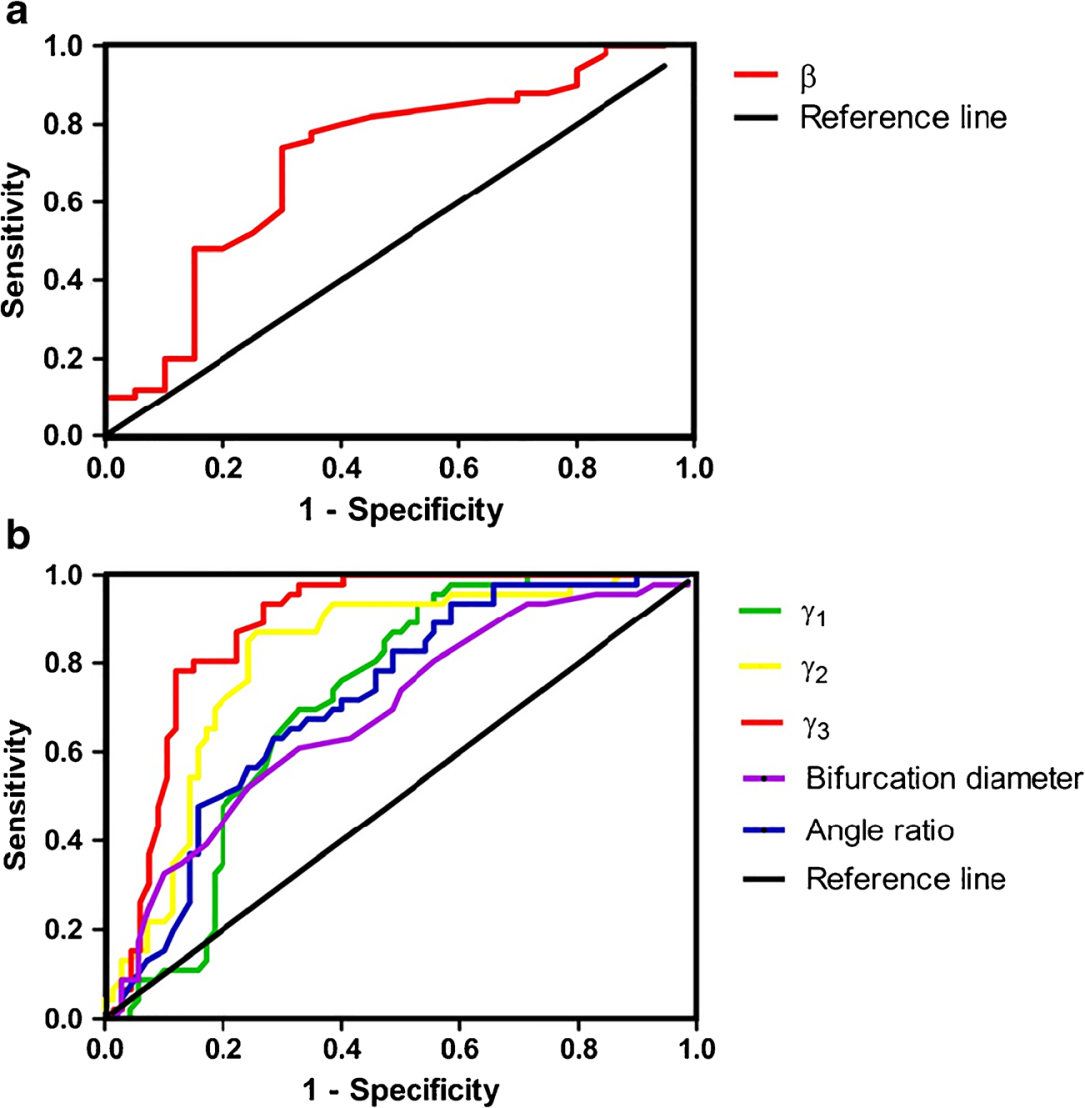
Receiver operator curves are plotted as a comparison of diagnostic efficacy for the measured vascular morphological parameters. **a** *β*. **b** *γ*_1_, *γ*_2_, *γ*_3_, the bifurcation diameter, and the angle ratio

The ROC curve analysis (Table 4, Fig. 9) results showed that the AUC of *γ*_1_, *γ*_2_, *γ*_3_, the bifurcation diameter in the MCA bifurcation aneurysm group, and *β* in the MCA distal aneurysm group were all greater than 0.7. Meanwhile, the cut-off value of *γ*_3_ = 104.50° had the highest diagnostic accuracy (AUC = 0.881), with a sensitivity of 78.26% and a specificity of 88.57%.

**Table 4:** Comparison of diagnostic efficacy of various vascular morphological parameters

	Cutoff score	Sensitivity(%)	Specificity(%)	AUC	95%CI
MCA distal aneurysms					
*β*(°)	113.50	72.00	70.00	0.713	0.571~0.854
MCA bifurcation aneurysms					
γ_1_(°)	103.50	93.48	47.14	0.717	0.624~0.809
*γ*_2_ (°)	98.00	86.96	74.29	0.812	0.731~0.893
*γ*_3_ (°)	104.50	78.26	88.57	0.881	0.818~0.945
Bifurcation diameter(mm)	2.45	60.87	67.14	0.720	0.623~0.812
Angle ratio	1.63	93.48	41.43	0.686	0.587~0.784

## Discussion

As early as 1930, scholars proposed that intracranial arterial morphology and hemodynamic changes were extremely important factors in the formation and development of aneurysms. Aneurysm geometry and hemodynamics interact with each other through blood flow (i.e., wall shear stress (WSS) and blood pressure) to determine the future geometry of an aneurysm (enlargement and morphological change) [15]. In this study, we aimed to study the correlation between the formation of MCA aneurysms and morphological parameters.

### Morphological characteristics of MCA bifurcation aneurysms and the contro

Several previously published studies support the findings presented here. Hademeno et al. showed that the direction of blood flow in the bifurcation of the vessel deviates, and the impact force and shear force on the bifurcation are significantly increased [16]. Their study confirmed that pressure could be 2–3 times that of adjacent arteries with aneurysms. Can et al. studied the bifurcation diameter and bifurcation angle between 73 MCA bifurcation aneurysm cases and 37 controls. In agreement with our results, they showed that the bifurcation diameter in aneurysm patients was smaller than in controls, while the bifurcation angle was larger [17]. Jianping et al. found that the most relevant parameter for the formation of MCA bifurcated aneurysms was the bifurcation angle, and its cut-off value was 124.8° [18]. In our study, *γ*_3_ also had the highest diagnostic accuracy (AUC = 0.881), but the cut-off value was 104.5°, a discrepancy that may be due to research protocol differences. Baharoglu et al. found that when the MCA branch inclination angle increases, the blood vessels turn sharply, thus deviating the inflowing blood and potentially leading to the formation of aneurysms [19]. Similarly, *γ*_1_ and *γ*_2_ were also associated with the formation of MCA aneurysms in our study. This may be due to the expansion of the bifurcation angle and the MCA branch inclination angle causing the WSS and wall shear stress gradient (WSSG) of vessels to increase.

Previous studies have explored that MCA bifurcation aneurysm formation is related to the angle of bifurcation, but whether it is related to *α* and *β* has rarely been reported. At present, only Duan et al. showed that *α* in the MCA bifurcation aneurysm group was smaller than the control group (mean 130° ± 15° vs. 133° ± 12°, *P* = 0.01) [20]. But in our study, there was no statistical difference in *α* between groups. At the same time, we found that *β* was not associated with the formation of bifurcation aneurysms. We speculate that the large distance between *α*, *β*, and the MCA bifurcation may have little effect on the blood flow pressure of the bifurcation. Thus, there is no obvious correlation with the formation of a bifurcation aneurysm.

The morphological parameters of the MCA bifurcation also include the vascular diameter. Smaller diameter vessels may be subjected to relatively strong hemodynamic stress, or the walls of smaller vessels may be too thin to withstand the said hemodynamic stress; both of these factors lead to the formation of aneurysms. Alexandra et al. showed that smooth blood vessels in patient models gradually thinned, which also led to the acceleration of blood flow at the MCA bifurcation, increased WSS and WSSG at the bifurcation vertex, and resulted in the formation of bifurcation aneurysms [21]. Bor et al. measured the geometric parameters of 104 cases of MCA bifurcation vessels and found that the incidence of aneurysms was higher in branch dysplasia (diameter < 1 mm) [22]. This agrees well with findings which point to the diameter cut-off value of 2.45 mm.

### Morphological features between MCA proximal and distal aneurysms and the control

A majority (80% to 85%) of MCA aneurysms occur at the bifurcation [23]. MCA proximal and distal aneurysms are relatively rare, and most previous studies of proximal and distal aneurysms focus on the selection of treatment options for aneurysm rupture. Few studies exist on the relationship between MCA geometry and the formation of proximal and distal aneurysms [24–27]. To the best of our knowledge, the relation of both the *α* and *β* to the formation of proximal and distal MCA aneurysms has not yet been reported. We compared and analyzed *α* and *β* between an MCA proximal aneurysm group, distal aneurysm group, and control group and found that there was no significant correlation between proximal aneurysm formation and *α* and *β*, while *β* in the distal aneurysm group was smaller than in controls. This difference was statistically significant (median 109.00° (19.00°) vs. 120.00° (19.25°), *P* = 0.010). When the cut-off value of *β* was 113.5°, the sensitivity and specificity of diagnosis were highest. We speculate that this correlation may be related to hemodynamics, but due to the small sample size, further studies are needed. However, there was no significant correlation between *α* and MCA distal aneurysm formation.

### Limitations

One limitation of this study is its retrospective nature. Although we have shown that the formation of MCA aneurysms is related to geometric shape, this does not directly suggest causality. Whether a difference in vascular morphology precipitates hemodynamic changes which then leads to the formation of aneurysms or whether peripheral vascular morphology changes during the formation of aneurysms is still unknown. Probing this question will require further studies.

## Conclusion

Our study focused on the effects of morphological aspects of the formation of MCA aneurysms. *γ*_1_, *γ*_2_, *γ*_3_, angle ratios, and bifurcation diameters were the relevant factors for the formation of MCA bifurcation aneurysms. MCA distal aneurysm formation was associated with *β*. The above parameters can be used to evaluate the susceptibility of aneurysms to rupture, which would facilitate the screening of high-risk groups of aneurysms.

## Data Availability

Not applicable.
